# Editorial: Camelid farming, production, reproduction, health, and welfare

**DOI:** 10.3389/fvets.2025.1674595

**Published:** 2025-08-22

**Authors:** Asim Faraz, Carlos Iglesias Pastrana, Laura Menchetti, Barbara Padalino

**Affiliations:** ^1^Department of Livestock and Poultry Production, Bahauddin Zakariya University, Multan, Pakistan; ^2^The Andalusian Institute for Agricultural, Fishing, Food and Ecological Production Research and Training (IFAPA), Córdoba, Spain; ^3^School of Biosciences and Veterinary Medicine, University of Camerino, Camerino, Italy; ^4^Division of Animal Sciences, Department of Agricultural and Food Sciences, Alma Master Studiorum, University of Bologna, Bologna, Italy; ^5^Faculty of Science and Engineering, Southern Cross University, Lismore, NSW, Australia

**Keywords:** dromedary camels, animal welfare, milk, genetics, one welfare

The Food and Agriculture Organization (FAO) year 2024 was dedicated to camelids (1), recognizing their growing role both as livestock and companion animals. To celebrate with FAO these species, this Research Topic entitled “*Camelid farming, production, reproduction, health, and welfare*” encompasses a broad spectrum of scientific disciplines, from milk production and reproduction to health and genetics. This multidisciplinary approach underscores the imperative of the “One Welfare” approach—recognizing the interconnected wellbeing of animals, humans, and the environment (2)—and highlights the necessity to include camels within this paradigm, a species that has been historically overlooked in scientific research. Once known as the “ships of the desert,” camelids are now recognized as the “livestock of the future” due to their multipurpose role (3). This evolving livestock and cultural paradigm emerges amid a growing global camelid population, presenting both opportunities and challenges in breeding strategies, food security, and animal welfare.

It is therefore fitting that this Research Topic introduces the first comprehensive protocol for assessing camel welfare under nomadic pastoral conditions (Padalino and Menchetti), alongside presenting the results of its initial application in Pakistan (Padalino et al.). The welfare assessment protocol adopts the established framework used in the Welfare Quality^®^ and European Animal Welfare Indicators (AWIN) projects for evaluating the welfare of other livestock species, and it builds upon the pioneering work developed for camels reared under intensive systems (4). The protocol incorporates the “Four Principles” of animal welfare (i.e., Good Feeding, Good Housing, Good Health, and Appropriate Behavior) along with multi-level assessment, a standardized scoring system, and farm classification. However, substantial adaptations to the conventional structure of welfare assessment protocols were required to account for the specific characteristics of camelids and their management systems. The traditional herd-level assessment was combined with a comprehensive interview with the caretaker, and additional management- and animal-based indicators were introduced to address context-specific challenges. Padalino et al. applied this protocol to 510 camels managed by nomadic pastoralists in Pakistan, revealing that extensively managed animals achieved higher overall welfare scores than their intensively farmed counterparts, principally due to enhanced freedom of movement and richer social interactions (reflecting high scores in “Appropriate Behavior”). Nonetheless, critical welfare risks persisted even in pastoral systems, including seasonal variability in food availability, predators, and lack of shade and shelter. Moreover, access to adequate veterinary care was a significant concern, especially in remote regions. Regardless of the specific outcomes, the implementation of the protocol in Pakistan has demonstrated its value as an effective tool for identifying key animal welfare concerns. However, more data is needed to understand the welfare level of dromedaries kept for different purposes in other parts of the world.

Alongside the resilience of extensive farming systems, this Research Topic also highlights significant shifts in camel breeding practices, particularly with regard to milk production. In our Research Topic, the interest in camel milk emerges as multidimensional, with potential spanning its health benefits, the modernization of nutrition and milking practices, and advances in genetic selection. Its therapeutic potential is widely recognized by local communities, but scientific evidence is needed to substantiate these claims. In this Research Topic, Behrouz et al. examined the effects of camel milk on various indicators of inflammation and oxidative stress in a model of cigarette smoke -induced chronic obstructive pulmonary disease. Treatment with camel milk led to a reduction in total and differential white blood cell counts, serum levels of TNF-α, and malondialdehyde concentrations in both serum and various tissues of the animal models. At the same time, it increased the levels of antioxidant enzymes and thiol compounds. The evidence thus indicates that camel milk exerts anti-inflammatory effects, by modulating the immune response, and mitigates oxidative stress, by enhancing antioxidant defenses and protecting cells from free radical-induced damage. The same authors also propose avenues for refining both the experimental model and future lines of investigation. Nonetheless, their study reinforces the idea that scientific research can substantiate the reputation of camel milk as the “white desert gold.”

With an emphasis on both nutritional interventions and milking technologies, the papers of Faraz et al. and Atigui et al. published under this Research Topic provide crucial insights into the optimization of camel milk output. Faraz et al. carried out a 45-day pilot study to examine the effects of a postbiotic derived from Saccharomyces yeast supplementation on milk yield and composition of camels raised in a semi-intensive manner. As compared to other groups, the group with higher supplementation had the highest milk yield and fat content, indicating a significant improvement in milk yield, especially in camels receiving greater dosages of postbiotics. Atigui et al. approach camel milk production from an alternative perspective, focusing on machine milking. While promising for improving farm profitability, the implementation of this technique requires specific adaptations to conventional technologies due to the camel's unique physiology and behavior. By figuring out the vacuum level required to open the teat sphincter (VLOTS) and recording the anatomical reactions of the teats during machine milking, Atigui et al. investigated the mechanical components of camel milking concurrently. Only 42% of teats opened below 70 kPa, indicating significant intra- and inter-animal heterogeneity in teat responsiveness. The publications collectively highlight the significance of combined approaches—nutritional improvement and customized mechanical procedures—to raise dairy camel milk yield and animal welfare.

As expected, several studies of this Research Topic focused on biotechnology to enhance camel reproduction, one of the fields most studied in camel science (5). In particular, El-Sokary et al. compared the efficiency of vitrification protocols for camel oviductal isthmus aggregates, focusing on the effects of aggregate size, cryoprotectants, cryodevices, post-thaw viability, and sperm-binding capacity. The research team completed five different experiments to achieve optimal preservation outcomes, and cryopreserving oviduct cell aggregated up to 150 μm in diameter using a 7 M cryoprotectant concentration, and 0.25 mL straw cryodevices were recommended. The second study of Mahdy and Nasr Eldeen presents for the first time a case of a completely divided female genital tract in a she-camel (i.e., uterus didelphys). This article presents a summary of the literature in other species, and a unique and rare pictures of uterous didelphys in a she-camels. It is worth noting that apparently the genital tract seems normal, until the persistence of the median walls of the Müllerian ducts along their entire length is detected. The wall results in two cervices and two separate uterine bodies. As this pathology is congenital, it may impair fertility. More studies on selective breeding should be performed, considering the advances made in the genetics of this species.

Three studies in this Research Topic explore innovative genetic strategies to improve camel breeding, with a focus on adaptation, lactation performance, and locomotion. A comprehensive genomic analysis of Awarik dromedaries from southwestern Saudi Arabia uncovered 66 and 53 candidate selection regions through iHS and nSL scans, encompassing a total of 308 genes (Almathen). Selection signals were particularly strong on chromosomes 15 and 16, with a dense cluster on chromosome 15 overlapping the TRNAI-AAU gene. Additional hotspots were identified on chromosomes 3, 2, 7, and 14, as well as large regions on chromosomes 11 (200 kb) and 9 (325 kb). Functional annotation of highlighted genes—such as BAG5, septin 7, SLC13A1, PCED1B, BMPR1B, ZAR1, JAKMIP2, and NOTCH2—points to diverse biological roles including olfaction, immune regulation, insulin secretion, reproduction, and cellular signaling. These findings not only deepen our understanding of adaptive mechanisms in desert environments, but also underscore the genetic value of locally adapted populations for resilient breeding and conservation under climate pressure. Complementing these genomic insights, a second study introduced the CamelBell No. 1 SNP array, the first functional 1K liquid SNP chip developed specifically for lactation performance in Bactrian camels (Guo et al.). Using RNA-seq data from 125 lactating females, the researchers selected 1,002 informative loci and validated the chip in 24 individuals, achieving >99% SNP call rates and 100% genotyping consistency. The chip was further tested on 398 camels from six breeding areas in Northwest China, confirming a shared genetic basis for milk-related traits. This tool provides a robust technical foundation to accelerate marker-assisted selection and represents a scalable platform for modern dairy camel breeding. Addressing an uncharted domain, a third study (Pastrana et al.) applied curve-estimation regression and canonical discriminant analysis to link form and function in camel locomotion. The cubic model was identified as the most suitable mathematical function to represent camel locomotion. Angularity and mechanical forces at distal limbs, pelvis inclination, hump-to-body proportionalities, post-neutering effects, and the velocity–acceleration profiles of the scapula, shoulder, carpus, hip, and foot emerged as decisive predictors of gait proficiency. These traits govern weight absorption, elastic energy storage, and propulsion efficiency, while generic animal or environmental variables proved negligible—likely buffered by innate desert-adapted features such as pacing gait and broad foot pads. The resulting criteria inform selective breeding for leisure and racing performance and even open avenues for camel-assisted therapy. Taken together, these three studies underscore a growing paradigm shift: from descriptive to predictive camel breeding. Whether through genotyping tools or phenotype assessment, these methods offer pathways to improve camel productivity, functionality, and welfare while preserving adaptive genetic traits critical for sustainability in desert ecosystems.

At least but not last, the Research Topic includes novel articles addressing health issues. One notable contribution is the comprehensive investigation of subclinical mastitis in dairy camels by Jama et al. in Ethiopia. Mastitis is a well-known problem in dairy cattle, but its dynamics in camels have been less studied. In this survey of 244 lactating camels, the authors found a 10.6% prevalence of subclinical mastitis, detected by California Mastitis Test. This data suggests that about 1 in 10 she-camels in dairy herds may carry hidden udder infections. The dominant pathogen isolated was *Staphylococcus aureus*, followed by *Streptococcus agalactiae* and *S. dysgalactiae*. Worryingly, antibiotic sensitivity testing revealed that many isolates of these bacteria had limited susceptibility to common drugs—for instance, only 44.7% were susceptible to oxytetracycline and 36.7% to tetracycline. Such findings hint at emerging antimicrobial resistance in camel mastitis pathogens, likely exacerbated by imprudent antibiotic use. Indeed, interviews with herders revealed widespread use of traditional remedies or over-the-counter drugs without veterinary guidance. The study also pointed to basic hygiene issues in these semi-intensive farms: two-thirds of owners allowed calves and other livestock to commingle and did not practice proper udder hygiene during milking. The implication is clear: improving camel health requires not just treating infections, but raising awareness and management standards among camel owners. The authors conclude that combating camel mastitis will necessitate alternative therapies (given drug resistance), thorough herder training, and better farm hygiene practices. Parasitic diseases are another concern addressed in the Research Topic. An emerging parasitology study by Al-Shaebi et al. documented the presence of *Eimeria rajasthani*—a coccidian parasite—in dromedary camels in Saudi Arabia. Through morphological and molecular analysis, the researchers confirmed this specific *Eimeria* species, which historically was reported in Indian camels, now in Arabian herds. Coccidiosis in camels is often under-recognized, but as the authors note, it can cause severe diarrhea and weight loss, especially in young animals; adults tend to develop immunity from prior exposure. The identification of *E. rajasthani* in a new region underscores the need for vigilance in camel health management. It suggests that parasite ranges may be shifting or simply that diagnostic efforts are catching up. With improved diagnostic tools, veterinarians can better monitor and control such parasitic infections (e.g., via strategic deworming or coccidiostats in young camels), ultimately reducing morbidity in camel calves.

The papers included in this Research Topic highlight the growing multidisciplinary interest in the dromedary camel, pointing on the interconnection among camel productivity, health, and welfare, as has already happened in other livestock species. However, the unique physiological and ethological characteristics of camelids—along with their distinct production contexts—necessitate significant adaptations, both in scientific methodology and in practical applications. For this reason, our compilation should be seen not as a conclusion, but rather as a starting point for a line of research that holds considerable potential for future scientific and cultural advancement.
